# Endothelial Protein C Receptor and Pediatric Arterial Stroke

**DOI:** 10.4274/tjh.01488

**Published:** 2013-03-05

**Authors:** Nejat Akar, Afife Karabıyık, Gülhis Deda

**Affiliations:** 1 Ankara University, School of Medicine, Department of Pediatric Molecular Genetics, Ankara, Turkey; 2 Ankara University, School of Medicine, Department of Pediatric Neurology, Ankara, Turkey

**Keywords:** EPCR, A3, sEPCR, Pediatric stroke

## Abstract

**Objective: **The aim of this study was to investigate the endothelial protein C receptor (EPCR) gene A3 haplotype and plasma soluble EPCR (sEPCR) levels in Turkish pediatric arterial stroke patients.

**Materials and Methods:** We analyzed 44 pediatric arterial stroke patients and 75 healthy controls. Following DNA isolation, genotyping of the A3 haplotype was determined via PCR and RFLP. Additionally, fasting sEPCR levels were determined via ELISA.

**Results: **There wasn’t a significant difference in the sEPCR level between the control and patient groups, although the sEPCR level was higher in the patient group. We didn’t observe a difference in the distribution of the CC and CG/GG genotypes between the control and patient groups.

**Conclusion:** Further study on sEPCR levels at the onset of pediatric stroke is needed in order to reach a more definitive conclusion.

**Conflict of interest:**None declared.

## Abstract (tr)

**Amaç:** Çalışmanın amacı, Türk populasyonundaki pediatrik arteriyel inme hastalarındaki Endotelyal Protein C Reseptörü (EPCR) geni A3 Haplotipi ve plazma solubl EPCR (sEPCR) seviyesinin araştırılmasıdır.

**Yöntemler:** Bu çalışmayla 44 pediatrik arteriyel inme hastası ve 75 sağlıklı kontrol analiz edildi. DNA izolasyonunu takiben, PCR ve RFLP yöntemleri kullanılarak A3 haplotipi genotiplendirilmesi yapıldı ve ELISA aracılığıyla da sEPCR seviyeleri saptandı.

**Bulgular:** Sağlıklı kontrol ve hasta grubu arasında sEPCR seviyesi açısından istatistiksel olarak anlamlı bir fark bulunmamasına ragmen, pediatrik arteriyel inme hastalarına ait grupta sEPCR seviyesi daha yüksek saptanmıştır.

**Sonuç:** Daha etkili bir sonuca varabilmek için pediatrik inmede plazma EPCR seviyelerinin araştırılacağı çalışmalara ihtiyaç vardır

## INTRODUCTION

Although stroke is a rare event in childhood, it constitutes approximately 33% of all pediatric thrombosis cases [1]. Stroke results in significant long-term morbidity and mortality in children. Various prothrombotic disorders, particularly those affecting the physiological anticoagulant system, are associated with the risk of stroke [2]. Active protein C (APC) is a major natural anticoagulant that limits the progression of the coagulation cascade via photolytic degradation of factor Va (FVa) and factor VIIIa (FVIIIa). Endothelial protein C receptor (EPCR) contributes to the interaction between thrombin-thrombomodulin and PC, and in this way this complex increases activation of PC [3]. 

PC activation is enhanced approximately 20-fold in vivo when PC is bound to EPCR [4]. EPCR is not only a 46-kDa endothelial cell-specific type-I transmembrane protein, it also has a soluble form circulating in plasma [5,6]. The extracellular domain of EPCR is cleaved with a metalloprotease and the cleavage results in the formation of soluble EPCR (sEPCR) [7]. EPCR plays a role in regulating coagulation and inflammation. sEPCR binds to PC and APC with the same affinity as transmembrane EPCR [8]. Furthermore, sEPCR inhibits the anti-inflammatory, profibrinolytic, and anticoagulant effects of APC. 

Elevated sEPCR is a risk factor for thrombosis [9]. EPCR is primarily expressed in the endothelium of large blood vessel [10]. EPCR is involved in numerous hemostatic, vascular, and immunological actions [8,11,12,13]. To date, >90 polymorphisms and 5 mutations of the human EPCR gene have been reported [14,15,16]. The EPCR A3 haplotype in exon 4, rs867186 (p.ser219gly; A4600G) is associated with increased plasma levels of sEPCR and thrombosis [17,18]. There is a strong association between the A3 haplotype and an elevated sEPCR level; an elevated sEPCR level might increase the risk of pediatric stroke [12]. The aim of the present study was to investigate the EPCR gene A3 haplotype and plasma sEPCR level in Turkish pediatric arterial stroke patients. 

## MATERIALS AND METHODS

The study included 44 pediatric arterial stroke patients (20 girls 24 boys) and 75 healthy controls (51 girls and 24 boys) that had never had thrombosis. Patients with perinatal strokes were excluded. All of the patients in the patient group had arterial, large vessel stroke. The control and patient groups were selected from among the data in our DNA bank for the period 1998-2011. Mean age in the control group was 29.4 years (because of the contents of our DNA bank) and mean age in patient group is 5.57 years. The control group was selected from among a group aged >18 years of age with no history of stroke. 

Measurement of plasma sEPCR in the patient group was performed 6 months after the first episode of stroke. Pediatric stroke patients with neonatal onset were evaluated for sEPCR after the age of 1 year [12]. Informed consent was obtained from the all the patients or their parents. The study protocol was approved by the Ethics Committee [13]. sEPCR levels were measured via enzyme-linked immunosorbent assay (ELISA) (Diagnostica Stago Asserachrom sEPCR, Asnieres-France). sEPCR levels 38-132 ng µL–1 were accepted as normal, according to our laboratory’s standards. 

Genomic DNA was extracted and the frequency of the EPCR gene A3 haplotype 1651 C-G was screened via PCR using the primers 5^′^-GCTGAAATTTTGTATTCTGTCC-3^′^ and 5^′^-CCAGTATAATGGCTACATTTTACC-3^′^, and annealing at 54 ^°^C. C1651G substitution creates a Eco91 I restriction site. The 293-bp PCR products were incubated using Eco91 I restriction enzyme (Fermentas, Lithuania) at 37 ^°^C for 16 h, and then the digested products were electrophoresed on 3% agarose gel (Sigma, USA). Additionally, sequencing of this region of the gene was performed (Beckman Coulter CEQ 8000, Beckman Coulter, USA). 

## RESULTS

The chi-square test, Student’s t test, Fisher's exact test, and P values were used for statistical analysis. Comparison of continuous variables between the patients and controls was based on Student's t test. The relationship between sEPCR and the A3 haplotype was determined using both Pearson’s and Spearman’s rank correlation analysis. The chi-square test was used to compare the observed frequencies using the Hardy-Weinberg equilibrium prediction. Statistical significance was set at p<0.05.

The frequency of the EPCR gene A3 haplotype (rs867186, C1651G, p.ser219gly) was determined (Table 1). There wasn’t a significant difference in the sEPCR level between the controls (n=75; mean sEPCR: 110.3±68.0) and patients (n=44; mean sEPCR: 90.8±62.6) (p> 0.05). In all, 34 of the pediatric arterial stroke patients carried 1651CC (mean sEPCR: 80.0±25.0), and 10 carried CG (CI = 0.67 [0.2-1.6]) and GG (CI = 4.7 [0.18-119]) (mean sEPCR: 213.3±67.5). There was a comparatively significant difference at the patient group in terms of their sEPCR levels and together with the A3 haplotype of the EPCR gene (p<0.0001). Patients with the CG/GG genotype had higher plasma sEPCR levels than those with the CC genotype. Similarly, the mean sEPCR level in controls with the 1651CG/GG genotype (n=21; mean sEPCR: 60.1±28.8) was significantly higher than in those with 1651CC (n =54; mean sEPCR: 161.0±62.8) (p=0.000). Although there wasn’t a significant difference in the distribution of the CC and CG/GG genotypes in the patient or control groups according to the chi-square test and Fisher’s exact test (p>0.05), the correlation between distribution of the sEPCR level and A3 haplotype, and the CC and CG/GG genotypes was significant (p= 0.01) based on Pearson’s and Spearman’s correlation analysis (Table 1).

## DISCUSSION

Several EPCR gene variations are accepted as risk factors for the development of thrombosis, because they cause a reduction in receptor expression or loss of receptor function. One such variation is a polymorphism referred to as haplotype 3. The EPCR A3 haplotype encodes serine instead of glycine at codon 219, which is located in the transmembrane region of EPCR [19]. One study reported that among patients with venous thrombosis the incidence of this polymorphism was higher than in healthy individuals [17]. On the other hand, individuals carrying the homozygous A3 haplotype have a 3-fold greater risk of coronary heart disease [18] and venous thrombosis [9]. The EPCR A3 haplotype is responsible for 86.5% of the variation in the sEPCR level that was generated via ADAM17 cleavage [9,19]. 

In the present study there wasn’t a significant difference in the sEPCR level between the control and patient groups, although the sEPCR level was higher in the patient group. Furthermore, CG/GG genotype carriers had higher plasma sEPCR levels than carriers of the CC genotype in both the patient and control groups. We did not observed a difference in the distribution of the CC and CG/GG genotypes between the control and patient groups. Further study on sEPCR levels at the onset of pediatric stroke is needed in order to reach a more definitive conclusion. As pediatric stroke is rare and its diagnosis at the onset of clinical symptoms is difficult, we think it would be beneficial to analyze sEPCR levels in adult stroke patients, as such data are lacking. 

**Conflict of Interest Statement**

The authors of this paper have no conflicts of interest, including specific financial interests, relationships, and/ or affiliations relevant to the subject matter or materials included.

## Figures and Tables

**Table 1:**
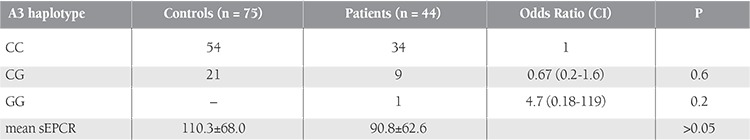
Distribution of the A3 haplotype and the mean sEPCR level in the control and patient groups.

## References

[1] Akar N (2002). Molecular approach to Turkish pediatric stroke patients. Turk J Hematol.

[2] Riela AR, Roach ES (1993). Etiology of stroke in children. J Child Neurol.

[3] Mosnier LO, Zlokovic BV, Griffin JH (2007). The cytoprotective protein C pathway. Blood.

[4] Taylor FB Jr, Peer GT, Lockhart MS, Ferrell G, Esmon CT (2001). Endothelial cell protein C receptor plays an important role in protein C activation in vivo. Blood.

[5] Zecchina G, Bosio S, Brusa E, Rege-Cambrin G, Camaschella C (2002). EPCR 23 bp insertion in a patient with severe progressive arterial disease: a dominant loss of function mutant in conditions of increased APC request. Br J Haematol.

[6] Kurosawa S, Stearns-Kurosawa DJ, Hidari N, Esmon CT (1997). Identification of functional endothelial protein C receptor in human plasma. J Clin Invest.

[7] Xu J, Qu D, Esmon NL, Esmon CT (1999). Metalloproteolytic release of endothelial cell protein C receptor. J Biol Chem.

[8] Esmon CT (2006). The endothelial protein C receptor. Curr Opin Hematol.

[9] Van Marion V, Rosendaal FR, Vos HL, Visser MC, Bertina RM (2004). Haplotypes of the EPCR gene, plasma sEPCR levels and the risk of deep venous thrombosis. J Thromb Haemost.

[10] Laszik Z, Mitro A, Taylor FB Jr, Ferrell G, Esmon CT (1997). Human protein C receptor is present primarily on endothelium of large blood vessels: implications for the control of the protein C pathway. Circulation.

[11] Akar N, Gokdemir R, Ozel D, Akar E (2002). Endothelial cell protein c receptor (EPCR) gene exon iii, 23 bp insertion mutation in the Turkish pediatric thrombotic patients. Thromb Haemost.

[12] Ulu A, Gunal D, Tiras S, Egin Y, Deda G, Akar N (2007). EPCR gene A3 haplotype and elevated soluble endothelial protein c receptor (sEPCR) levels in Turkish pediatric stroke patients. Thromb Res.

[13] Yururer D, Teber S, Deda G, Egin Y, Akar N (2009). The Relation Between Cytokines, Soluble Endothelial Protein C Receptor, and Factor VIII Levels in Turkish Pediatric Stroke Patients. Clin Appl Thromb Hemost.

[14] Esmon CT (2000). The endothelial cell protein C receptor. Thromb Haemost.

[15] Medina P, Navarro S, Estelles A, Vaya A, Bertina RM, Espana F (2005). Influence of the A4600G and C4678G polymorphisms in the endothelial protein C receptor (EPCR) gene on the risk of venous thromboembolism in carriers of factor V Leiden.. Thromb Haemost.

[16] Franchi F, Biguzzi E, Cetin I, Facchetti F, Radaelli T, Bozzo M, Pardi G, Faioni EM (2001). Mutations in the thrombomodulin and endothelial protein C receptor genes in women with late fetal loss. Br J Haematol.

[17] Saposnik B, Reny JL, Gaussem P, Emmerich J, Aiach M, Gandrille S (2004). A haplotype of the EPCR gene is associated with increased plasma levels of sEPCR and is a candidate risk factor for thrombosis. Blood.

[18] Karabiyik A, Yilmaz E, Egin Y, Akar N (2012). The effects of Endothelial Protein C Receptor Gene Polymorphisms on sEPCR levels in Venous Thrombotic Patients. Turk J Hematol.

[19] Saposnik B, Lesteven E, Lokajczyk A, Esmon CT, Aiach M, Gandrille S (2008). Alternative mRNA is favored by the A3 haplotype of the EPCR gene PROCR and generates a novel soluble form of EPCR in plasma. Blood.

